# Abnormal resting-state EEG neural oscillations and functional connectivity in mild cognitive impairment

**DOI:** 10.3389/fnagi.2025.1640966

**Published:** 2025-09-12

**Authors:** Yi Jiang, Zhiwei Guo, Rubing Jiao, Haoru He, Ning Jiang, Jiayuan He

**Affiliations:** ^1^The National Clinical Research Center for Geriatrics, West China Hospital of Sichuan University, Chengdu, Sichuan, China; ^2^The Med-X Center for Manufacturing, Sichuan University, Chengdu, Sichuan, China; ^3^West China Biomedical Big Data Center, West China Hospital, Sichuan University, Chengdu, Sichuan, China

**Keywords:** mild cognitive impairment, MCI, electroencephalography, rest-stage, functional connectivity

## Abstract

**Background:**

Mild cognitive impairment (MCI) exhibits abnormal resting-state EEG oscillations in delta (1–4 Hz), theta (4–7 Hz), and alpha (8–13 Hz) bands, though findings remain inconsistent. Moreover, dynamic functional connectivity (FC) alterations in these bands are poorly understood. To address this, we aimed to characterize resting-state EEG oscillations and dynamic FC in these frequency bands in MCI.

**Method:**

We recruited 21 MCI and 20 age−/education-matched normal controls (NC). Resting-state EEG was recorded for 5 min (eyes-open). We utilized power spectral density to investigate abnormalities in neural oscillations, and employed the directed transfer function (DTF) to explore dynamic functional connectivity (FC) alterations within the delta, alpha, and theta frequency bands 4among two groups.

**Results:**

Compared to NC, for neural oscillation, MCI showed significantly increased delta oscillation (prefrontal, parietal, temporal, and central regions) mainly located in the frontal and parietal lobes, significantly decreased alpha oscillation of the entire brain region mainly located in the frontal lobe, and both significantly increased and decreased theta oscillation (prefrontal, parietal, and occipital lobes) with fewer electrodes. For dynamic brain FC, in the delta band, the MCI exhibited significantly enhanced bidirectional FC between the prefrontal and parietal lobes, as well as two bottom-up FC from the occipital lobe to the central and parietal regions; In the theta band, the MCI showed significant enhancement of two FC from the temporal lobe to the frontal lobe, two FC from the occipital lobe to the parietal lobe, and one FC from the parietal lobe to the frontal lobe; In the alpha band, the MCI had one significantly enhanced bottom-up FC from the occipital lobe to the prefrontal lobe.

**Conclusion:**

During the eyes-open resting-state, differences of two groups in neural oscillations were primarily observed in the alpha and delta bands. The MCI exhibited significantly decreased alpha oscillations in the frontal lobe and increased delta oscillations in the frontal and parietal lobes. However, dynamic FC differences were most prominent in the delta and theta bands, including significantly increased interconnectivity of the prefrontal parietal network and significantly increased bottom-up FC. These findings emphasize the necessity of comprehensive analysis of local activity and large-scale network dynamics in MCI.

## Introduction

Alzheimer’s disease (AD) stands as the primary etiology of dementia, accounting for an approximate range of 60–80% of all dementia cases (Palmqvist et al., 2025). It causes a gradual decline in cognitive function, which in turn leads to a decreased or even lost ability to live independently, work, and participate in society (Alzheimer’s Association Report, 2023). AD represents a seamless continuum of pathological progression, initiating with subtle, asymptomatic biological changes—such as the accumulation of amyloid-beta (Aβ) plaques—and steadily advancing toward overt clinical manifestations. Within this spectrum, mild cognitive impairment (MCI) is characterized as the earliest identifiable stage marked by quantifiable cognitive deficits, rather than merely serving as a transient intermediary between asymptomatic and symptomatic phases. At this juncture, individuals with MCI already exhibit neuropathological hallmarks of AD, including Aβ plaques and neurofibrillary tangles of tau protein, yet their cognitive decline remains below the threshold required for a dementia diagnosis (Bruno et al., 2024; Jack et al., 2024). Although some MCI patients may remain stable or even revert to normal cognition, MCI remains a major risk factor for AD or other forms of dementia (Jongsiriyanyong and Limpawattana, 2018). Given that AD currently has no cure, early identification of MCI-AD could help mitigate cognitive decline or delay disease progression. In cases where active AD pathology is present in MCI, it may lead to alterations in cortical dynamics, which could be detected through the analysis of neurophysiological data (Das and Puthankattil, 2020). Therefore, investigating objective and reliable biomarkers to identify individuals at high risk of AD conversion represents a promising research direction.

Currently, multiple diagnostic systems are employed for MCI detection, including neuroimaging, magnetoencephalography (MEG), positron emission tomography (PET), cerebrospinal fluid (CSF) and blood-based biomarkers (e.g., amyloid-β42/40 ratio, phosphorylated tau, and neurofilament light chain). However, due to their high costs and invasive nature, the continuous application of neuroimaging techniques, MEG, and PET in MCI clinical trials may face limitations—particularly in low- and middle-income countries (Babiloni et al., 2020a). Furthermore, with the exception of FDG-PET as an indirect marker of synaptic integrity, existing biomarkers fail to reflect the impact of MCI neuropathology on the neurophysiological transmission of neural signals that underpin cognitive processes. To address this gap, resting-state electroencephalography (rsEEG) rhythm oscillations have emerged as a highly promising alternative. rsEEG offers several advantages including non-invasiveness, test–retest reliability (without practice effects until severe dementia stages), cost-effectiveness, and global accessibility based on widely available recording technologies (Babiloni et al., 2020b). It enables the investigation of MCI’s effects on ascending activating systems and reciprocal thalamocortical circuits, where oscillatory synchronization signals across brain regions dynamically regulate cortical arousal during quiet vigilance (Rossini et al., 2020; Pfurtscheller, 1989). Such phase synchronization/desynchronization of cortical neural activity may occur in an interrelated manner across multiple cortical areas, governing the transmission and communication of action potentials within local and long-range neural networks (de Haan et al., 2009; Stam et al., 2007; Pievani et al., 2011). Animal studies have elucidated the cellular and molecular basis of sustained EEG activity in cortical and subcortical regions (Buzsáki et al., 2013; Crunelli et al., 2015), demonstrating that AD neuropathology may lead to neuronal disconnectivity, impaired cortico-cortical and cortico-subcortical pathways, and myelinated axon loss. These alterations could be associated with cortical neuronal hyperexcitability, hypersynchrony, as well as reduced neurotransmission, neural signaling, and synaptic activity (Ahnaou et al., 2019; Shah et al., 2016). EEG devices can capture spontaneous rhythmic neural electrical activity. Under standard clinical and research conditions, EEG signals are typically divided into five typical frequency bands: *δ* (1–4 Hz), *θ* (4–7 Hz), *α* (8–13 Hz), *β* (13–30 Hz), and *γ* (30–100 Hz). Existing reviews on rsEEG studies in MCI and AD indicated that patients exhibit abnormalities in posterior α and widespread δ and θ rhythms, including deviations in peak frequency, power, and “connectivity,” which correlate with disease progression and interventions (Babiloni et al., 2021). Despite these conclusive summaries, a significant gap remains in applying these frequency-band findings to clinical diagnosis and intervention. This stems from heterogeneity in study populations, variations in eyes-open vs. eyes-closed resting-state paradigms, and methodological inconsistencies. Moreover, controversies persist regarding abnormal brain regions across frequency bands. For instance, in an European DESCRIPA rsEEG study, compared to non-amnestic MCI, preclinical AD without amyloid deposition, and age-matched controls, AD-MCI exhibited increased fronto-occipital *θ* and reduced posterior *α* power (Babiloni et al., 2010). Conversely, another study found that AD and AD-MCI, relative to controls, displayed higher θ power density in temporal and parieto-occipital regions, alongside lower α power density in parieto-occipital areas and reduced β2 power density in frontal and temporal lobes—all linked to cognitive deficits (Roh et al., 2011). Thus, for MCI, a frequency-band-specific analysis of abnormal neural oscillations across brain regions during the resting state is highly valuable, as it can help elucidate some of the existing controversies.

EEG not only captures local neural oscillations but also enables the measurement of correlations in neural activity between distinct brain regions through sophisticated non-linear dynamic analyses, thereby elucidating the brain’s functional integration capacity. Current research has demonstrated that MCI is associated with abnormal functional integration, conceptualized as a disconnection syndrome, suggesting that cognitive impairments may primarily stem from disrupted communication between brain regions rather than deterioration within discrete neural systems. For instance, task-based studies indicate that individuals with MCI exhibit impaired functional connectivity between temporal and prefrontal regions during memory encoding, accompanied by increased complexity in brain network organization, ultimately contributing to memory deficits (Jiang et al., 2025; Jiang et al., 2024). Review articles further reveal that both AD and MCI patients display significant alterations in functional and effective connectivity within resting-state brain networks, particularly in the *α* and *θ* frequency bands (Paitel et al., 2025). These changes reflect diminished network integration capacity and are closely associated with cognitive dysfunction. For instance, AD and MCI patients exhibit markedly weakened α-band functional connectivity (Stam, 2014), while θ-band connectivity more frequently demonstrates hyperconnectivity in these populations (Sheline and Raichle, 2013). However, existing resting-state studies based on brain network analysis report inconsistent findings (Stam et al., 2007; de Haan et al., 2009; He et al., 2008; Yao et al., 2010; Miraglia et al., 2016; Abazid et al., 2021). Most research focuses on α and θ bands, while only a limited number of investigations address abnormalities in the brain network associated with the *δ* band. Moreover, conventional analytical approaches predominantly employ non-directional, phase-based frequency-domain metrics, which primarily reflect temporal synchronization of neural population activity between brain regions without inferring the causal directionality of information flow (Cohen, 2014). Current causal analysis methods, also referred to as effective connectivity analyses, include granger causality, directed coherence, and directional transfer function (DTF). Among these, DTF as a frequency-domain effective connectivity analysis method offers distinct advantages in frequency specificity, temporal resolution, multivariate analysis capability, noise robustness, visualization of causal pathways, and relevance to neurophysiological mechanisms (Kamiński and Blinowska, 1991; Ding et al., 2000; Kuś et al., 2004; Blinowska, 2011). Therefore, DTF-based dynamic network analysis across *δ*, *θ*, and *α* frequency bands can reveal abnormal functional connectivity patterns in MCI, providing deeper insights into aberrant information processing mechanisms within neural networks of AD and MCI patients.

Neural oscillations and interregional connectivity exhibited distinct characteristics in terms of functional specificity, spatial scale, and temporal dynamics across different frequency bands, yet remain interconnected through frequency-band coupling and network integration to collectively support brain functional networks. Since neural oscillations and interregional connectivity are two key components, they are both interrelated and distinct. An independent analysis of the two would overlook the dynamic interactive nature between them. Therefore, it is necessary to establish an integrated analytical framework that encompasses neural oscillations across all frequency bands in various brain regions, as well as whole-brain dynamic network connectivity, to comprehensively understand the aberrant patterns in MCI during the resting state. In summary, our objectives were: (1) to investigate regional neural oscillation abnormalities in *δ*, *θ*, and *α* bands in MCI using resting-state EEG; and (2) to examine dynamic network connectivity abnormalities in δ, θ, and α bands in MCI through rsEEG dynamic network analysis.

## Materials and methods

### Participants

Given that MCI is not easily detectable in the community environment, this study first conducted a public awareness campaign through posters in Min’an Community, Chengdu, aiming to enhance residents’ understanding of MCI, and then proceeded with the recruitment of participants for the research. The assessment scales employed included: the Mini-Mental State Examination (MMSE), Montreal Cognitive Assessment (MoCA), Subjective Cognitive Decline Self Rating Scale (SCD-21), Activities of Daily Living scale (ADL), Geriatric Depression Scale (GDS), Generalized Anxiety Disorder scale (GAD), and Ischemic Scale.

Given the relatively low sensitivity of the MMSE in detecting MCI, particularly among individuals with higher educational attainment (Zhang et al., 2021), and considering that all participants in this study were recruited from highly educated communities, reliance solely on MMSE may result in underdiagnosis of MCI. In our study, an MMSE score > 24 can swiftly rule out potential patients with very early-stage mild dementia. The MoCA demonstrates superior sensitivity for subtle cognitive deficits and is more appropriate for MCI diagnosis (Ciesielska et al., 2016; Jia et al., 2021). Combining MMSE and MoCA through a dual-threshold strategy (MMSE >24 + MoCA <26) can enhance diagnostic accuracy, ensuring enrollment of individuals with mild yet measurable cognitive impairment but not meet the dementia criteria. Similar criteria have been validated in prior MCI research to balance sensitivity and specificity, particularly in highly educated populations (Julayanont and Nasreddine, 2017). However, it is noteworthy that the diagnosis of MCI generally relies on clinical evaluation, neuropsychological testing, as well as reports from patients and their caregivers, rather than solely on scores of cognitive assessment scale (Petersen et al., 2018; Petersen, 2016).

Inclusion criteria for MCI participants: met the diagnostic guidelines of the 2018 Chinese Dementia and Cognitive Impairment Diagnosis and Treatment Standards; Self-reported memory complaints; MoCA score <26/30 with MMSE score >24; Instrumental Activities of Daily Living score >6/8 and ADL = 100; Age 65–75 years; No use of anti-Alzheimer’s medications; No history of neurological or psychiatric disorders; No history of diabetes mellitus; No history of cardiac disease; Compliance with informed consent protocols.

Inclusion criteria for cognitively normal controls (NC): no self-reported memory complaints; MoCA score >26/30 and MMSE score >24; Age 65-75 years; No psychiatric or central nervous system disorders; No history of diabetes mellitus; No history of cardiac disease; Compliance with informed consent protocols.

### Demographic characteristics of participants

We enrolled a total of 41 elderly participants from Chengdu’s Min’an community, comprising 21 individuals with MCI and 20 NC. The research protocol was approved by the Ethics Committee of West China Medical School, Sichuan University. All participants provided written informed consent prior to enrollment. The baseline characteristics of participants are presented in Table 1.

**Table 1:** Descriptive statistics of the NC group and the MCI group represent as mean (SD).

Category	NC group *N* = (20)	MCI group *N* = (21)	*t/x^2^*	*p*
Sex	Female (*N* = 12) Male (*N* = 8)	Female (*N* = 15) Male (*N* = 6)	0.595	0.44
Age (years)	70.00 (3.39)	70.33 (2.85)	0.444	0.659
Education (years)	11.05 (3.63)	9.52 (3.50)	1.369	0.179
MOCA	26.70 (1.38)	19.91 (2.98)	9.284	<0.01^*^
MMSE	28.05 (1.43)	26.76 (2.02)	2.343	0.024^*^

SD, standard deviation; NC, cognitively normal elderly; MCI, mild cognitive impairment; MOCA, Montreal Cognitive Assessment; MMSE, Mini Mental State Examination. *p < 0.05.

### EEG data acquisition and pre-processing

The EEG recordings were conducted in an electrically shielded room. A NeuroScan system was used to acquire 32-channel EEG signals (Electrodes placed according to the international 10–20 system) at a sampling rate of 250 Hz. During recording, electrode impedance was maintained below 10 kΩ. Participants remained seated with eyes open in front of a blank computer screen while minimizing movement. Five minutes of resting-state EEG data were collected from per participant.

The EEG Data Pre-processing involved the following key steps: Average Reference: The raw signals were re-referenced to the common average reference. Bandpass filtering: A 1–30 Hz bandpass filter (Butterworth, zero-phase) was applied to remove low-frequency drifts and high-frequency noise. Independent Component Analysis (ICA): Artifact removal was performed using the Infomax algorithm in EEGLAB to identify and eliminate noise components (ocular and muscular artifacts). Baseline correction and epoch segmentation: The continuous 5-min EEG recording was segmented into 60 non-overlapping 5-s epochs.

### Power spectral density analysis

Power Spectral Density (PSD) quantifies the power distribution of neural oscillations across frequency bands. Given established findings that resting-state EEG activity primarily localizes to *δ* (1–4 Hz), *θ* (4–7 Hz), and *α* (8–13 Hz) bands, we computed each electrode’s PSD values within these frequency ranges using Welch’s method:


(1) $$P_{l}(W)=\frac{1}{n}{|\sum_{m=0}^{n-1}\varepsilon (m).e^{-\mathrm{jwm}}|}^{2}$$

where j denotes the imaginary unit, W represents frequency, n indexes EEG samples, and *x*(*n*) corresponds to each channel’s time series. Equation 1 yields PSD values for all channels in δ, θ, and α bands.

### Dynamic brain network construction

We implemented DTF to model causal brain networks. DTF characterizes directional information flow between multichannel signals based on Granger causality principles. A 32-channel multivariate autoregressive (MVAR) model was constructed:


(2) $$X(t)=[X_{1}(t),X2(t),\ldots ,\mathrm{Xi}(t)\ldots ,X32(t)]$$

The time series vector X(t) is defined as Equation 2, where X_i_ represents the *i*_th channel’s time series. The MVAR model is expressed as Equation 3:


(3) $$X(t)=\sum_{n=1}^{p}\mathrm{AnX}(t-n)+e(t)$$

Here, *A_n_* denotes 32 × 32 coefficient matrices, *e(t)* is white noise, and model order *p* = 2 was determined via Bayesian Information Criterion (BIC). Frequency-domain transformation yields Equation 4:


(4) $$X(f)=A^{-1}(f)e(f)=H(f)e(f)$$

The normalized DTF is defined as:


(5) $$\gamma_{\mathrm{ij}}^{2}(f)=|H_{\mathrm{ij}}(f)|2/\sum_{m=1}^{k}|H_{\mathrm{im}}(f)|2$$

In the Equation 5, γ^2^ij(f) represents the ratio of the normalized influence from channel j to channel i relative to the total influence from all channels to channel i. A higher value indicates a stronger causal relationship from the source channel j (cause) to the target channel i (effect), and vice versa, where k denotes the total number of channels.

Non-zero values suggest the existence of causal connectivity between channels j and i, though such connectivity may represent spurious correlations. To address this, we employed the surrogate data method proposed by Kaminski in 1991 to test the significance of effective functional connectivity, thereby identifying genuine connections and eliminating chance-induced false positives. The core principle of this method involves generating an empirical distribution for significance testing through the following procedure: First, the EEG signals from each channel were phase-randomized to create surrogate datasets. This randomization process was repeated 1,000 times to construct a null distribution of DTF values. The 950th sorted value (corresponding to the 95th percentile) served as the significance threshold (*α* = 0.05). When the actual DTF value exceeded this threshold, the causal functional connectivity from channel j to i was considered statistically significant (DTF > 0). Conversely, functional connectivity below the threshold were deemed non-significant (DTF = 0).

For each of frequency bands, we computed 32 × 32 DTF matrices where: Nodes represent EEG electrodes edges correspond to directional transfer function values between channels DTF_Mean denotes the frequency-averaged DTF value across the band of interest. As a network metric, DTF_Mean quantitatively characterizes connectivity patterns and serves as a direct indicator of causal functional connectivity strength within the network.

### Statistical analysis

Statistical analyses were performed using SPSS 22.0 software. In the analysis of two groups of continuous variables (Age, Education, MoCA, MMSE, behavioral measures, PSD, and DTF_Mean values), we employed the Shapiro–Wilk test to evaluate the normality of both groups and used Levene’s test to determine whether the variances of the two groups were equal. When the data met the assumptions of normality and homogeneity of variance, independent samples t-tests were conducted and Cohen’s *d* was calculated to quantify the effect size of group differences; otherwise, the Mann–Whitney *U* test was applied and Cliff’s delta was computed to estimate the magnitude of distributional difference. For the analysis of two groups of non-continuous variables (gender), we employ the chi-square test. Pearson correlation analysis was conducted to examine relationships between cognitive scale scores and DTF_Mean values. The false discovery rate (FDR) correction was applied for multiple comparisons of EEG metrics (PSD, and DTF_Mean values). The level of statistical significance was set at *p* < 0.05.

## Results

### PSD analysis results

**Figure 1:**
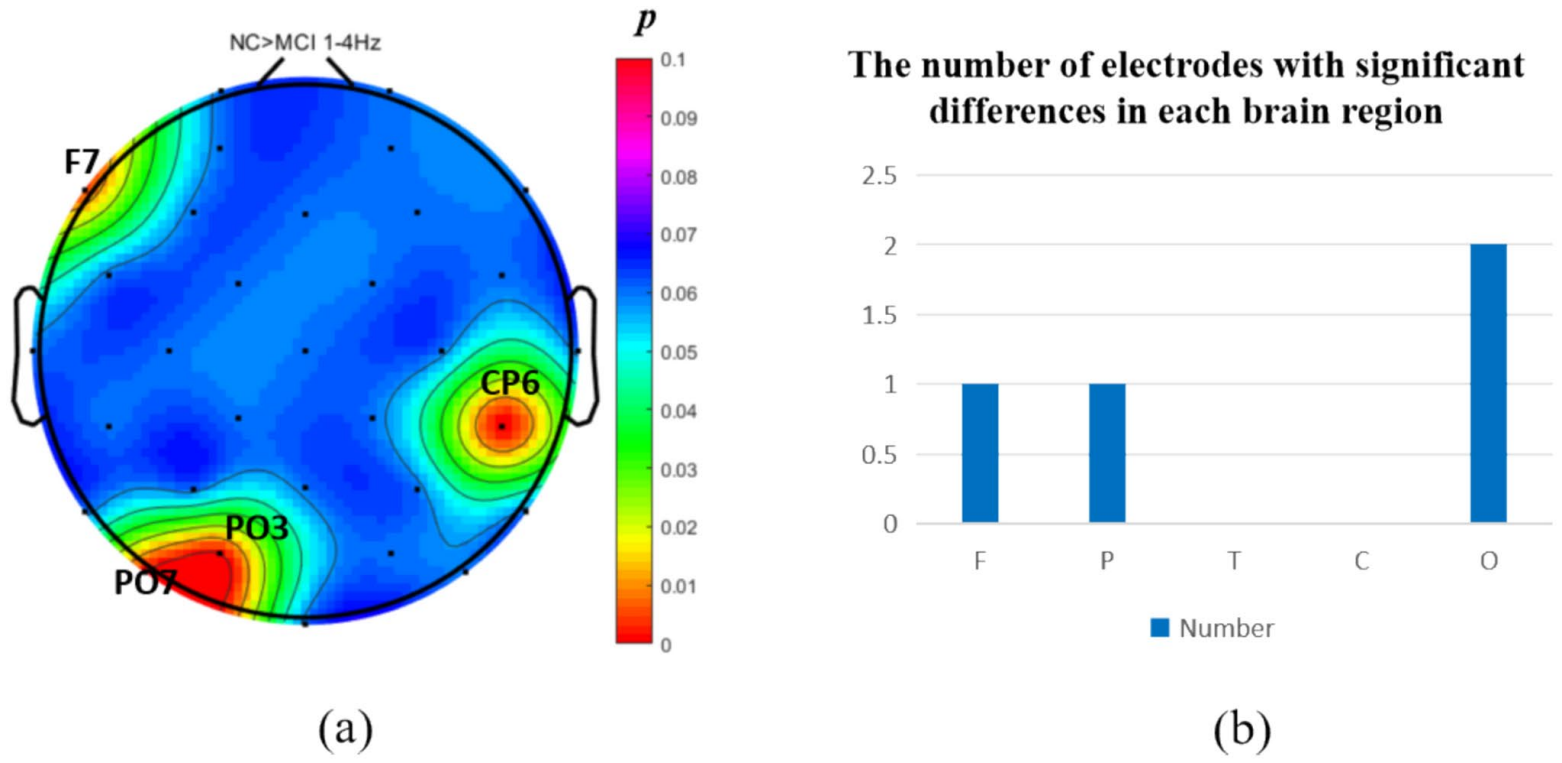
Statistical analysis of *δ* oscillation distribution between NC and MCI. **(a)** Distribution of electrodes with significant differences in brain regions. **(b)** Statistics on the numbers of electrodes with significant differences based on brain regions. NC > MCI. (*p* < 0.05) (FDR corrected). F, frontal lobe; O, occipital lobe; P, parietal lobe; T, temporal lobe; C, central district.

**Figure 2:**
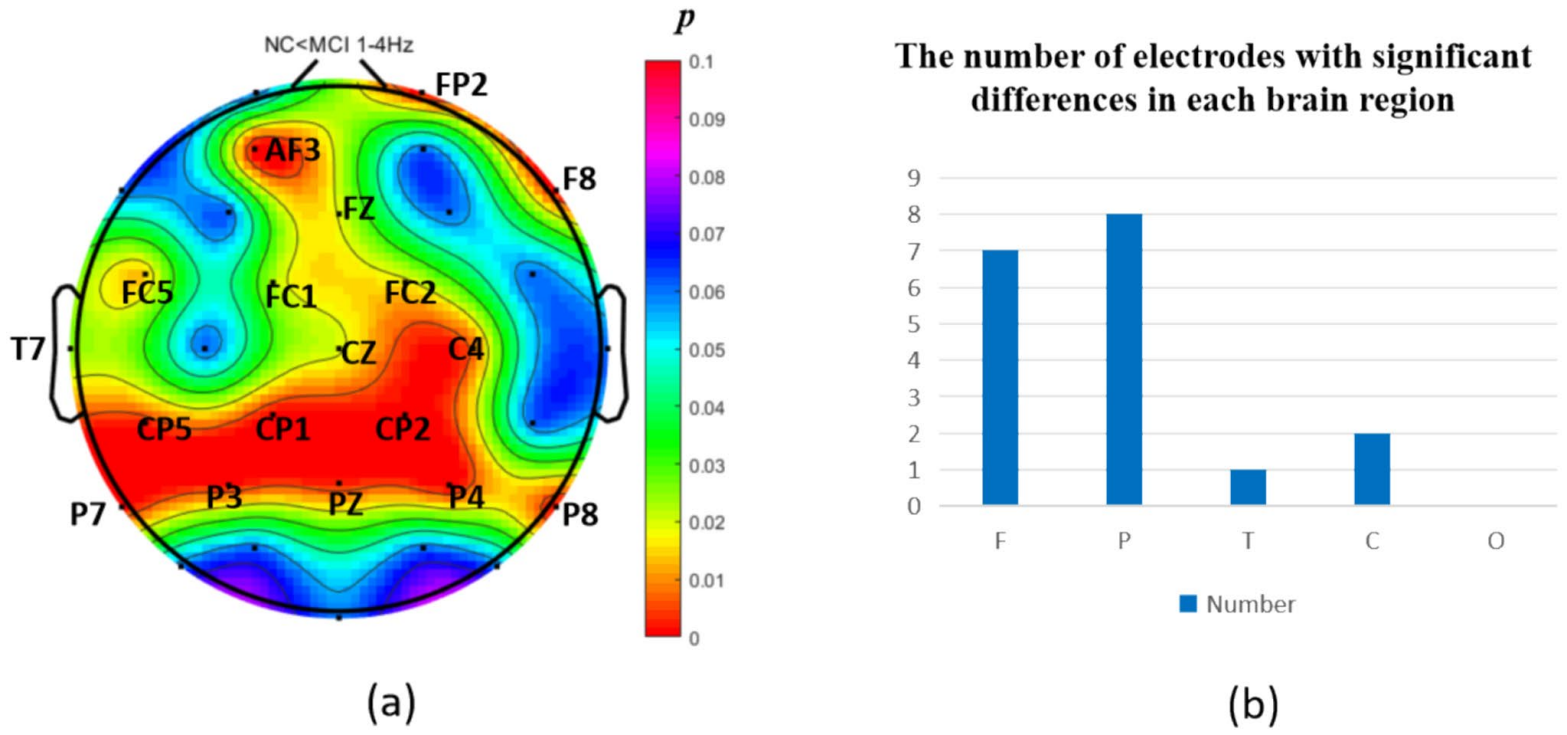
Statistical analysis of δ oscillation distribution between NC and MCI. **(a)** Distribution of electrodes with significant differences in brain regions. **(b)** Statistics on the numbers of electrodes with significant differences based on brain regions. NC < MCI. (*p* < 0.05) (FDR corrected). F, frontal lobe; O, occipital lobe; P, parietal lobe; T, temporal lobe; C, central district.

The analysis of neural oscillation differences in the *δ* band between groups revealed that the MCI group exhibited significantly decreased δ oscillations (*p* < 0.05) at 1 prefrontal electrode: F7 (effect size: −0.23), 1 parietal electrode: CP6 (effect size:-0.44), and 2 occipital electrodes: PO3, PO7 (effect size: −0.28; −0.27), totaling 3 brain regions (4 electrodes) as shown in Figure 1. Meanwhile, the MCI group demonstrated significantly increased δ oscillations (*p* < 0.05) at 7 prefrontal electrodes: F8, FP2, AF3, Fz, FC1, FC2, FC5 (effect size:0.22;0.27;0.21;0.20;0.20;0.21;0.21), 8 parietal electrodes: CP1, CP2, CP5, P3, P4, P7, P8, Pz (effect size:0.25;0.29;0.23;0.24;0.31;0.26;0.24;0.24), 1 temporal electrode: T7 (effect size:0.20), and 2 central electrodes: C4, CZ (effect size:0.22;0.20), totaling 4 brain regions (16 electrodes) as shown in Figure 2.

**Figure 3:**
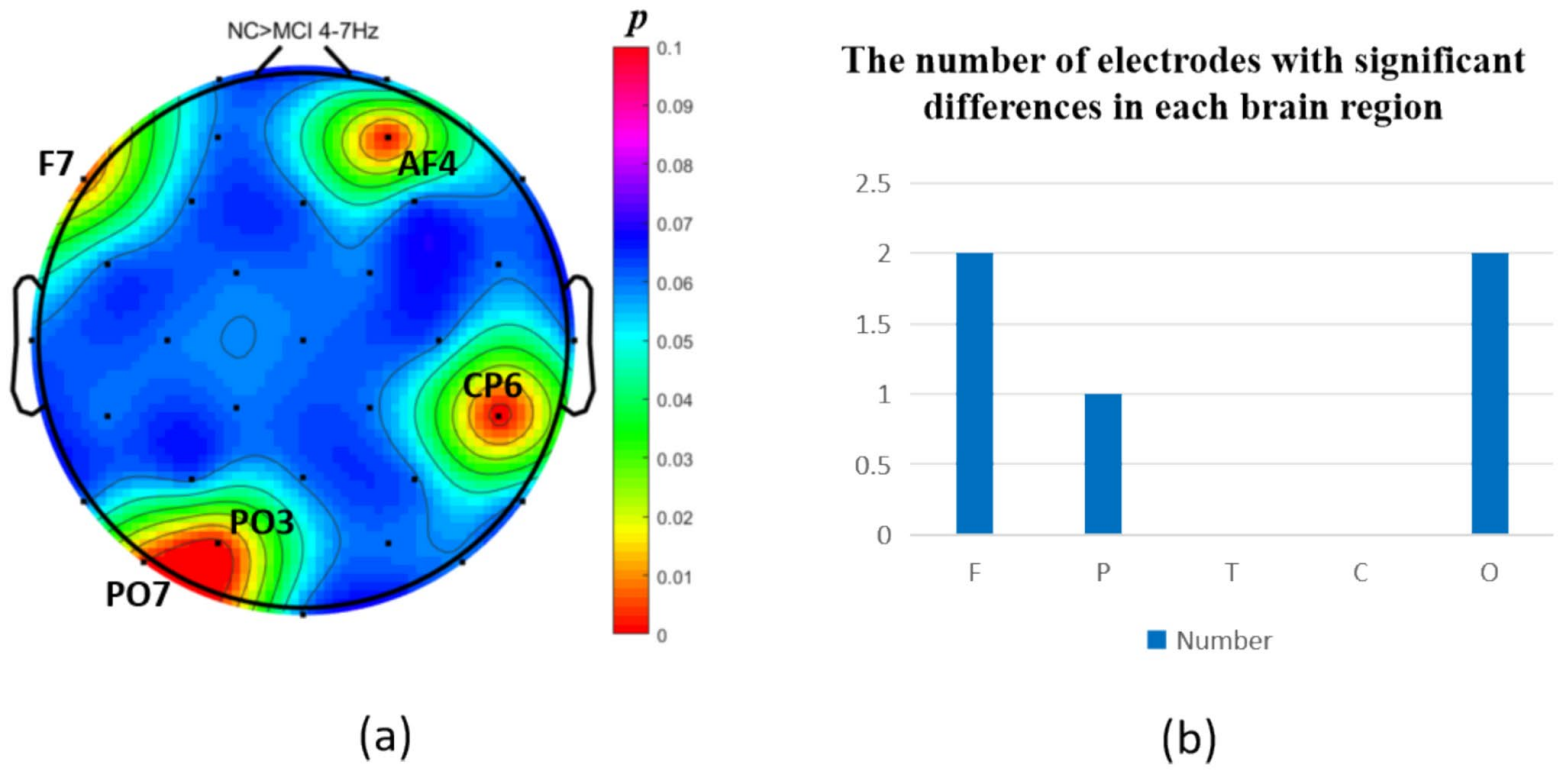
Statistical analysis of *θ* oscillation distribution between NC and MCI. **(a)** Distribution of electrodes with significant differences in brain regions. **(b)** Statistics on the numbers of electrodes with significant differences based on brain regions. NC > MCI. (*p* < 0.05) (FDR corrected). F, frontal lobe; O, occipital lobe; P, parietal lobe; T, temporal lobe; C, central district.

**Figure 4:**
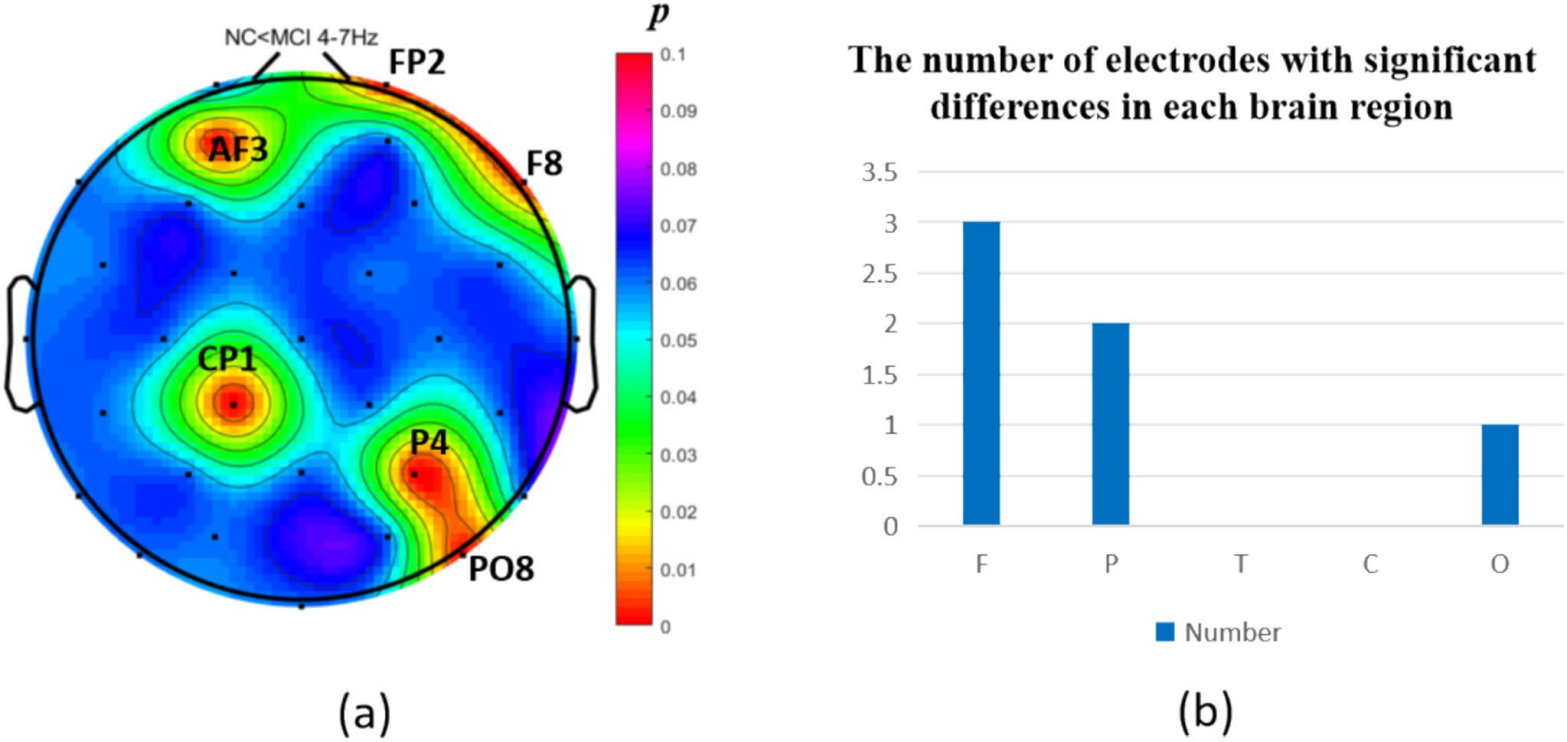
Statistical analysis of θ oscillation distribution between NC and MCI. **(a)** Distribution of electrodes with significant differences in brain regions. **(b)** Statistics on the numbers of electrodes with significant differences based on brain regions. NC < MCI. (*p* < 0.05) (FDR corrected). F, frontal lobe; O, occipital lobe; P, parietal lobe; T, temporal lobe; C, central district.

The intergroup analysis of *θ*-band neural oscillations demonstrated that the MCI group showed significantly decreased θ oscillations (*p* < 0.05) at 2 prefrontal electrodes: F7, AF4 (effect size: −0.22; −0.24), 1 parietal electrode: CP6 (effect size:-0.44), and 2 occipital electrodes: PO3, PO7 (effect size: −0.31; −0.33), totaling 3 brain regions (5 electrodes) as shown in Figure 3. Meanwhile, the MCI group exhibited significantly increased θ oscillations (*p* < 0.05) at 3 prefrontal electrodes: F8, FP2, AF3 (effect size: 0.25; 0.37;0.27), 2 parietal electrodes: CP1, P4 (effect size: 0.34; 0.33), and 1 occipital electrode: PO8 (effect size: 0.25), totaling 3 brain regions (6 electrodes) as shown in Figure 4.

**Figure 5:**
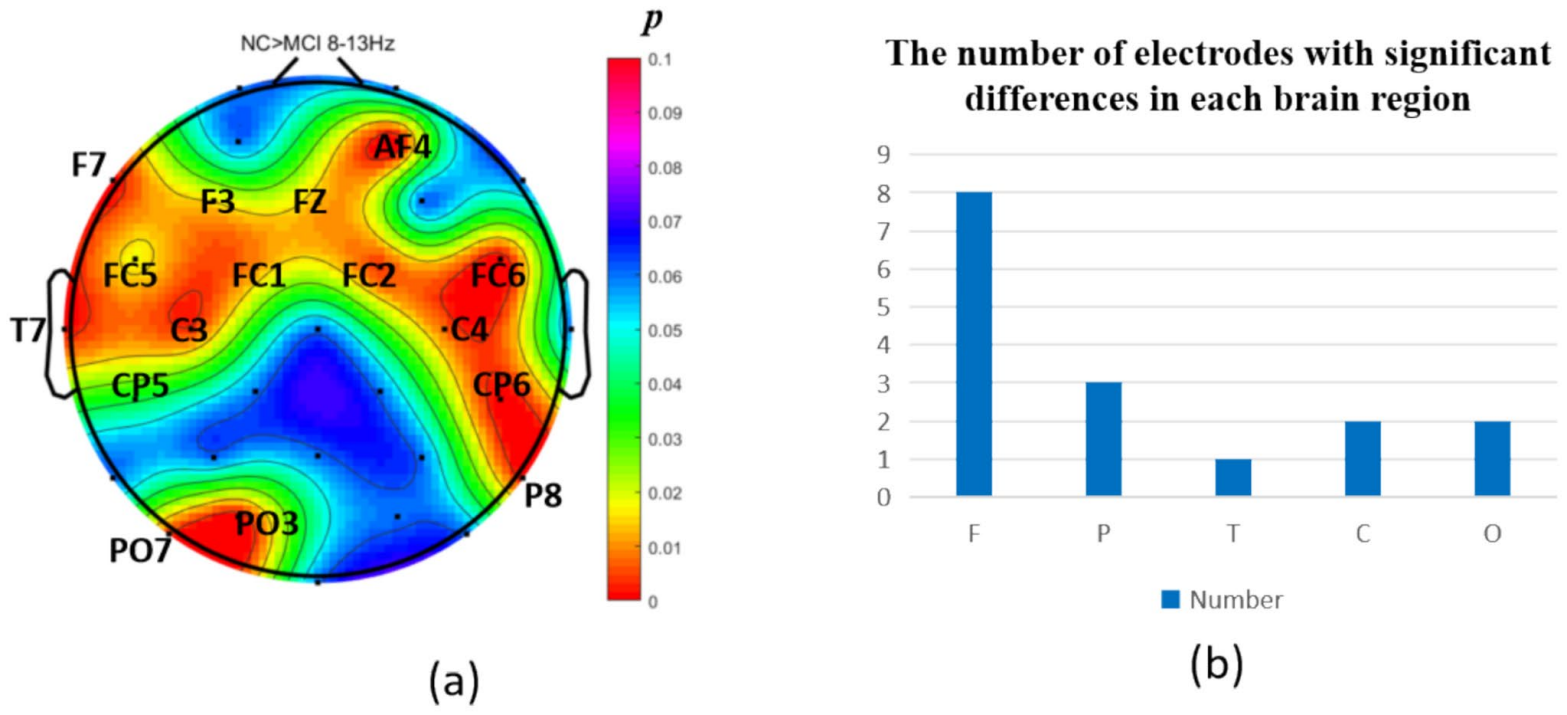
Statistical analysis of *α* oscillation distribution between NC and MCI. **(a)** Distribution of electrodes with significant differences in brain regions. **(b)** Statistics on the numbers of electrodes with significant differences based on brain regions. NC > MCI. (*p* < 0.05) (FDR corrected). F, frontal lobe; O, occipital lobe; P, parietal lobe; T, temporal lobe; C, central district.

**Figure 6:**
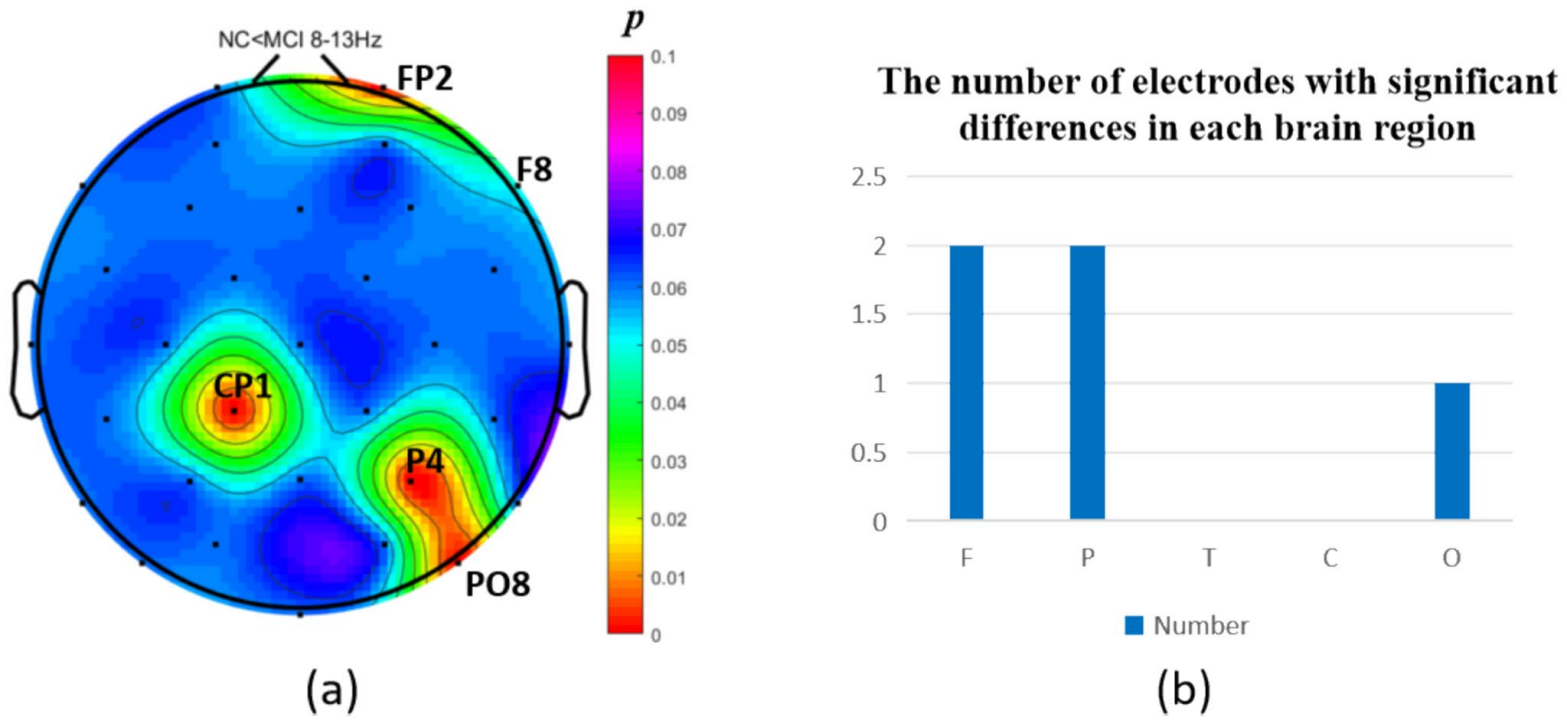
Statistical analysis of α oscillation distribution between NC and MCI. **(a)** Distribution of electrodes with significant differences in brain regions. **(b)** Statistics on the numbers of electrodes with significant differences based on brain regions. NC < MCI. (*p* < 0.05) (FDR corrected). F, frontal lobe; O, occipital lobe; P, parietal lobe; T, temporal lobe; C, central district.

The intergroup analysis of *α*-band neural oscillations revealed that the MCI group displayed significantly decreased α oscillations (*p* < 0.05) at 8 prefrontal electrodes: F7, F3, Fz, FC1, FC2, FC5, FC6, AF4 (effect size: −0.31; −0.21; −0.31; −0.34; −0.23; −0.21; −0.28; −0.31), 3 parietal electrodes: CP6, CP5, P8 (effect size: −036; −0.29; −0.34), 1 temporal electrode: T7 (effect size: −0.25), 2 central electrodes: C3, C4 (effect size: −0.23; −0.32), and 2 occipital electrodes: PO3, PO7 (effect size: −0.24; −0.33), totaling 5 brain regions (16 electrodes) as shown in Figure 5. Meanwhile, the MCI group showed significantly increased α oscillations (*p* < 0.05) at only 2 prefrontal electrodes: F8, FP2 (effect size: 0.29; 0.24), 2 parietal electrodes: CP1, P4 (effect size: 0.29; 0.21), and 1 occipital electrode: PO8 (effect size: 0.37), totaling 3 brain regions (5 electrodes), as shown in Figure 6.

### Results of resting-state dynamic network analysis

#### Intergroup analysis of resting-state dynamic network patterns

**Figure 7:**
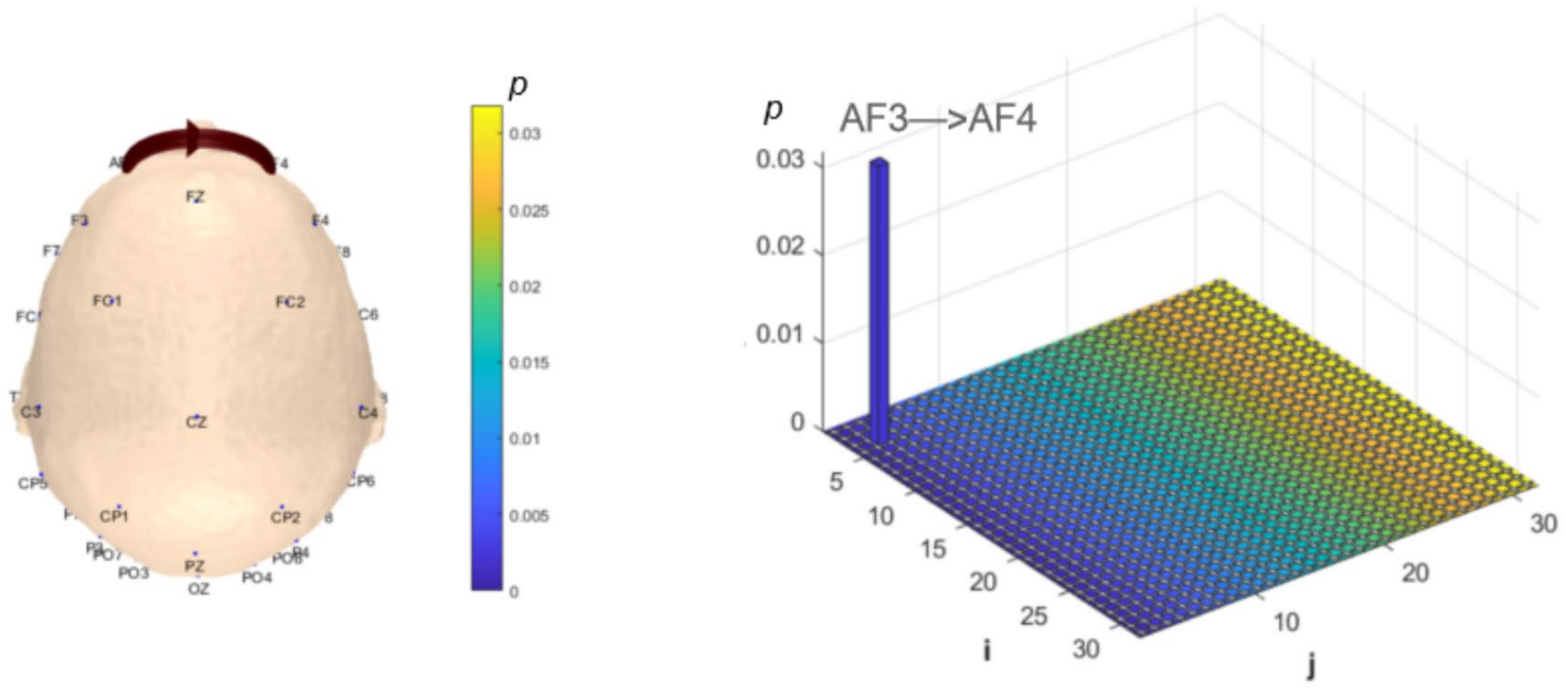
Statistical analysis of δ-band dynamic network connectivity between NC and MCI. The *i*-axis and *j*-axis represent 32 EEG electrodes respectively, with functional connectivity directed from *j* to *i* (NC > MCI, *p* < 0.05, FDR-corrected).

**Figure 8:**
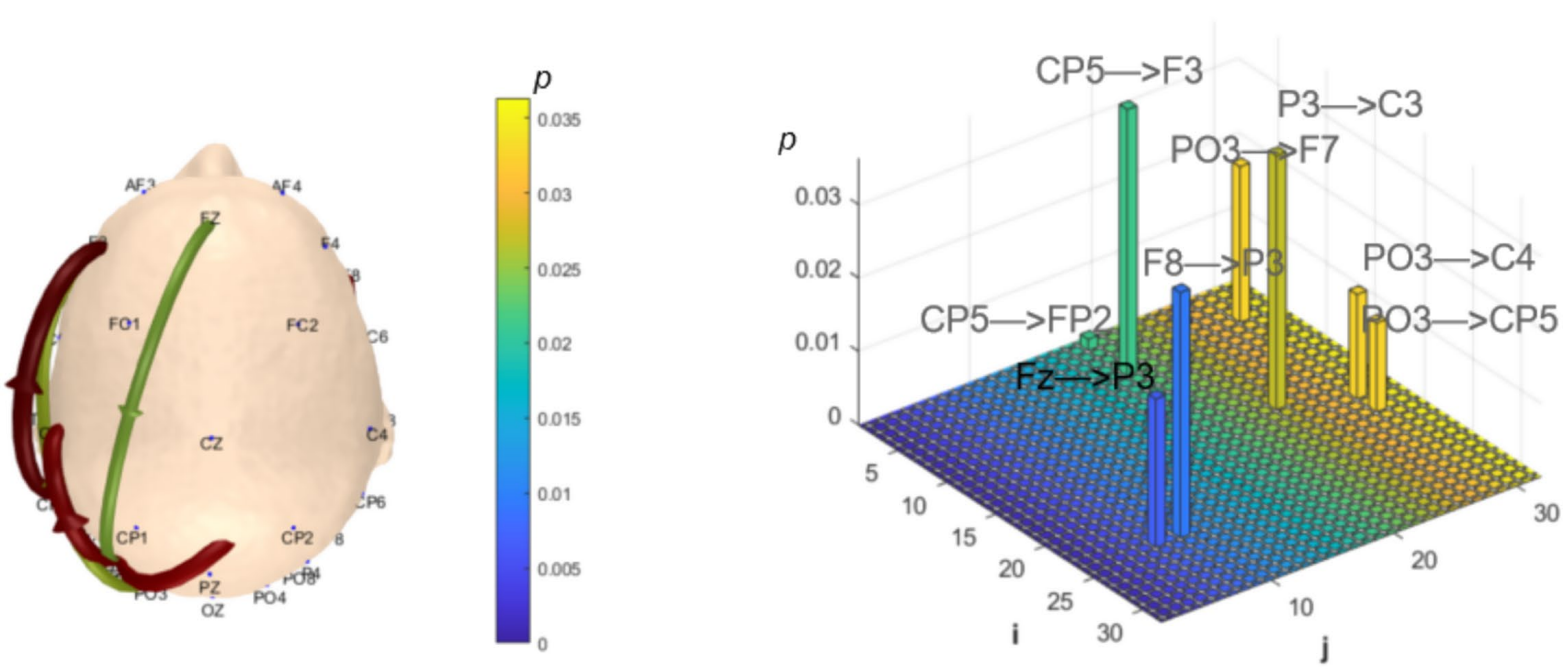
Statistical analysis of δ-band dynamic network connectivity between NC and MCI groups. The *i*-axis and *j*-axis represent 32 EEG electrodes respectively, with functional connectivity directed from *j* to *i* (NC < MCI, *p* < 0.05, FDR-corrected).

Functional connectivity and network topology between brain regions reflect information transfer and integration, representing the overall functional integration of the brain. Analysis of dynamic resting-state brain networks helps further explain the differences between local neural oscillations in brain regions and interregional functional connectivity. The intergroup analysis of *δ*-band dynamic network connectivity showed that the MCI group had significantly reduced prefrontal (AF3) to prefrontal (AF4) functional connectivity (number = 1, *p* < 0.05,effect size: −0.70), as shown in Figure 7. Meanwhile, the MCI group exhibited significantly enhanced top-down prefrontal (Fz, F8) to parietal (P3) functional connectivity (number = 2, *p* < 0.05, *d* = 0.76; effect size: 0.69), bottom-up parietal (CP5) to prefrontal (FP2, F3) functional connectivity (number = 2, *p* < 0.05, effect size: 1.06; 0.67), parietal (P3) to central (C3) functional connectivity (number = 1, *p* < 0.05, effect size: 0.68), and bottom-up occipital (PO3) to prefrontal (F7), central (C4), and parietal (CP5) functional connectivity (number = 3, *p* < 0.05, effect size: 0.75; 0.80; 0.82), as shown in Figure 8.

**Figure 9:**
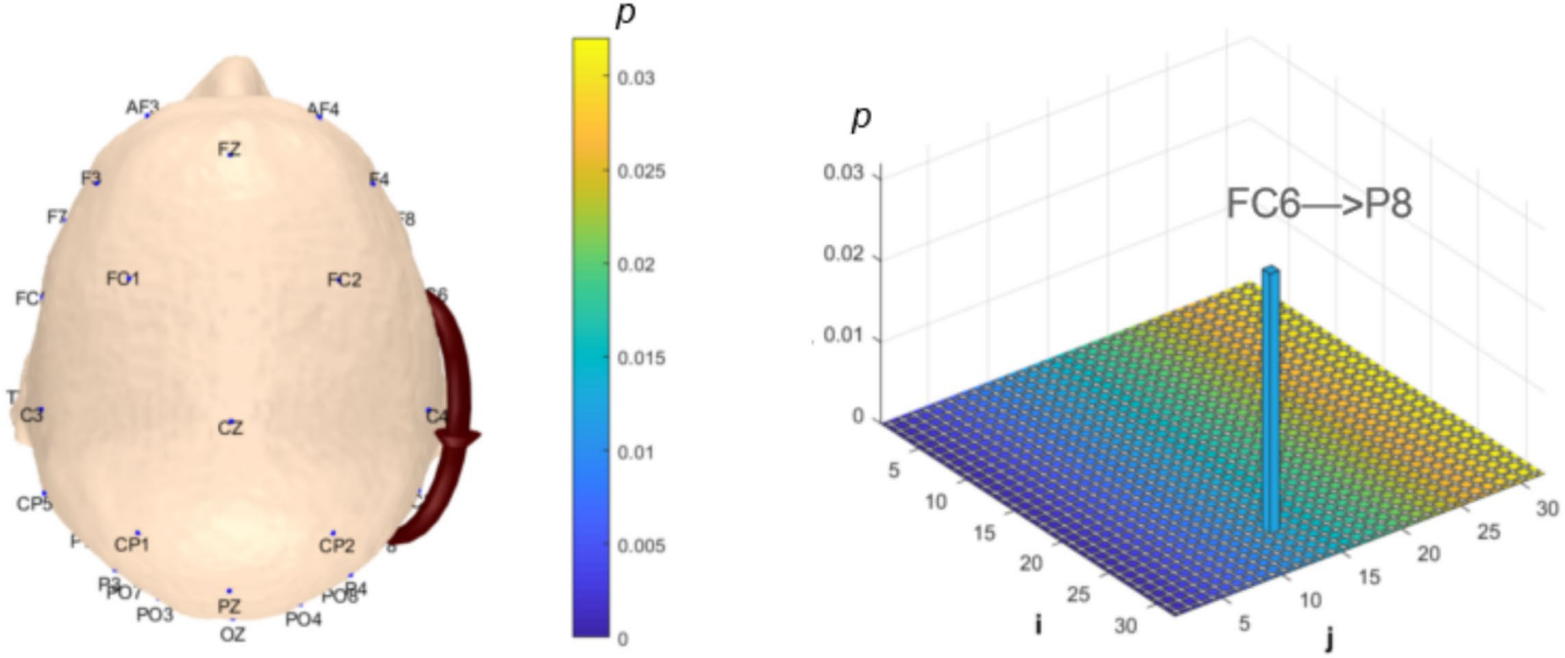
Statistical analysis of θ-band dynamic network connectivity between NC and MCI. The *i*-axis and *j*-axis represent 32 EEG electrodes respectively, with functional connectivity directed from *j* to *i* (NC > MCI, *p* < 0.05, FDR-corrected).

**Figure 10:**
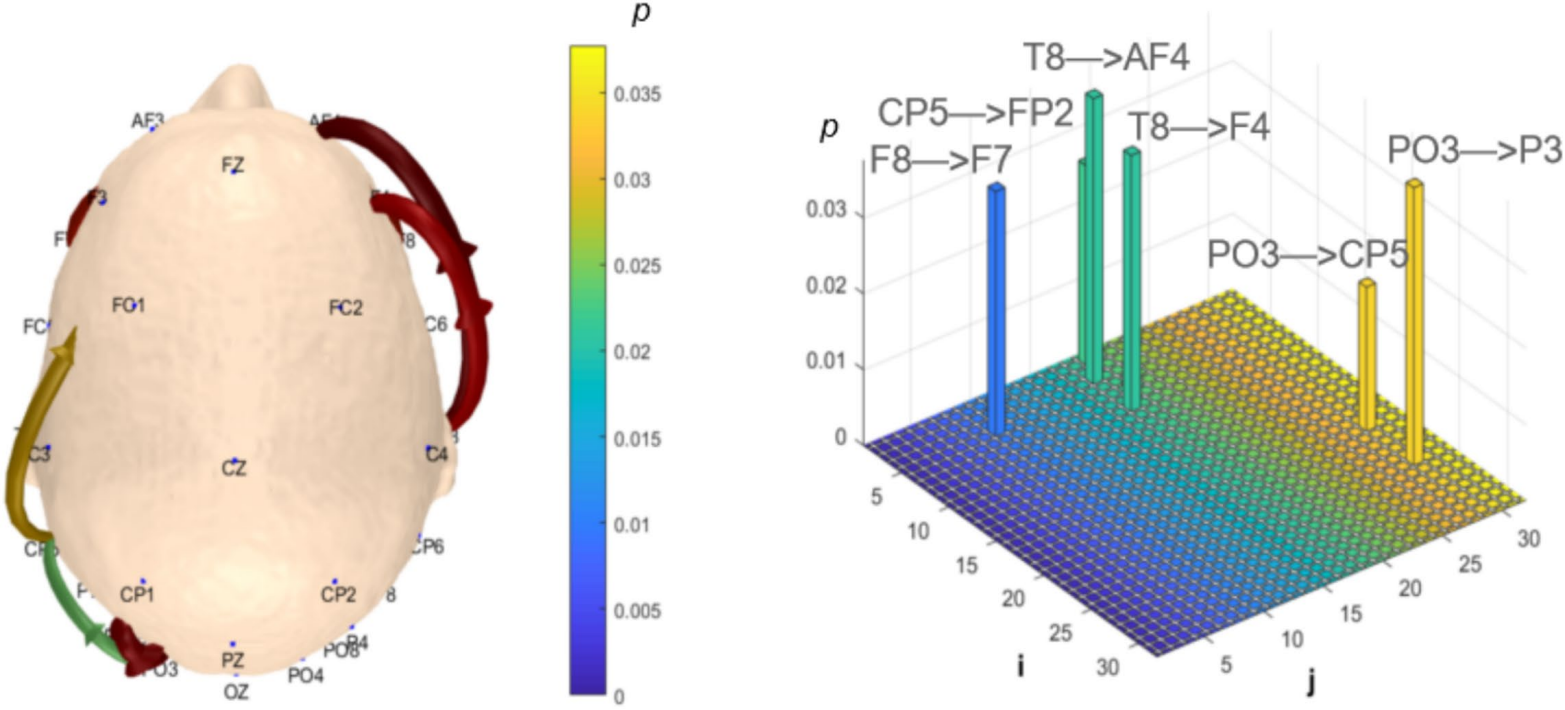
Statistical analysis of θ-band dynamic network connectivity between NC and MCI. The *i*-axis and *j*-axis represent 32 EEG electrodes respectively, with functional connectivity directed from *j* to *i* (NC < MCI, *p* < 0.05, FDR-corrected).

Analysis of *θ*-band dynamic network connectivity differences between groups revealed that the MCI group showed significantly reduced top-down functional connectivity from prefrontal FC6 to parietal P8 (number = 1, *p* < 0.05, effect size: −0.69), as shown in Figure 9. Meanwhile, the MCI group demonstrated significantly enhanced top-down temporal T8 to prefrontal AF4/F4 functional connectivity (number = 2, *p* < 0.05, effect size: 0.67; −0.69), occipital PO3 to parietal P3/CP5 functional connectivity (number = 2, *p* < 0.05, effect size: 0.68; −0.76), bottom-up parietal CP5 to prefrontal FP2 functional connectivity (number = 1, *p* < 0.05, effect size: 0.72), and prefrontal F8 to F7 functional connectivity (number = 1, *p* < 0.05, effect size:0.69), as shown in Figure 10.

**Figure 11:**
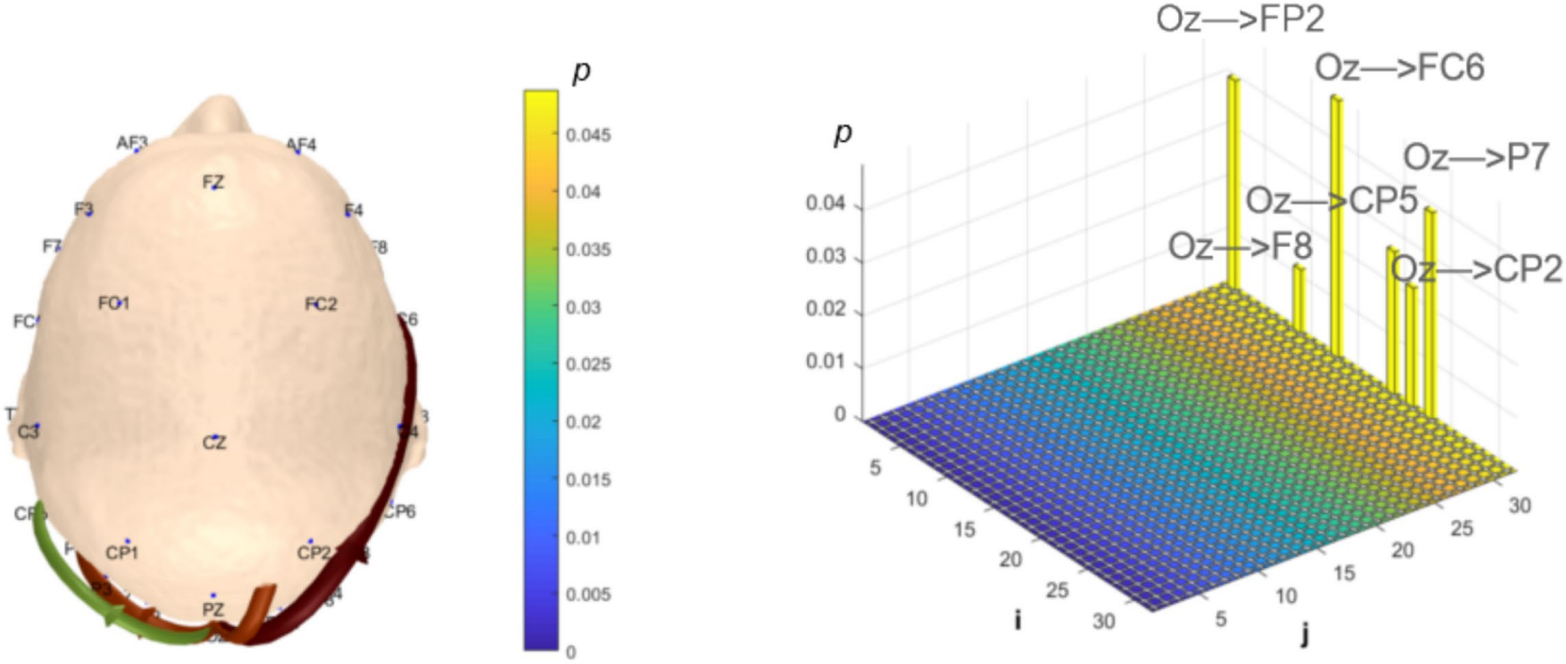
Statistical analysis of α-band dynamic network connectivity between NC and MCI. The *i*-axis and *j*-axis represent 32 EEG electrodes respectively, with functional connectivity directed from *j* to *i* (NC < MCI, *p* < 0.05, FDR-corrected).

Analysis of *α*-band dynamic network connectivity revealed no significantly reduced functional connectivity in the MCI group (*p* > 0.05). However, the MCI group exhibited significantly enhanced bottom-up occipital (Oz) to prefrontal (FP2, F8, FC6, P7) functional connectivity (number = 4, *p* < 0.05, effect size: 0.66; 0.82; 0.64; 0.67) and bottom-up occipital (Oz) to parietal (CP5, CP2) functional connectivity (number = 2, *p* < 0.05, effect size: 0.72; 0.74), as shown in Figure 11.

#### Correlation analysis between resting-state dynamic network connectivity and MoCA

**Figure 12:**
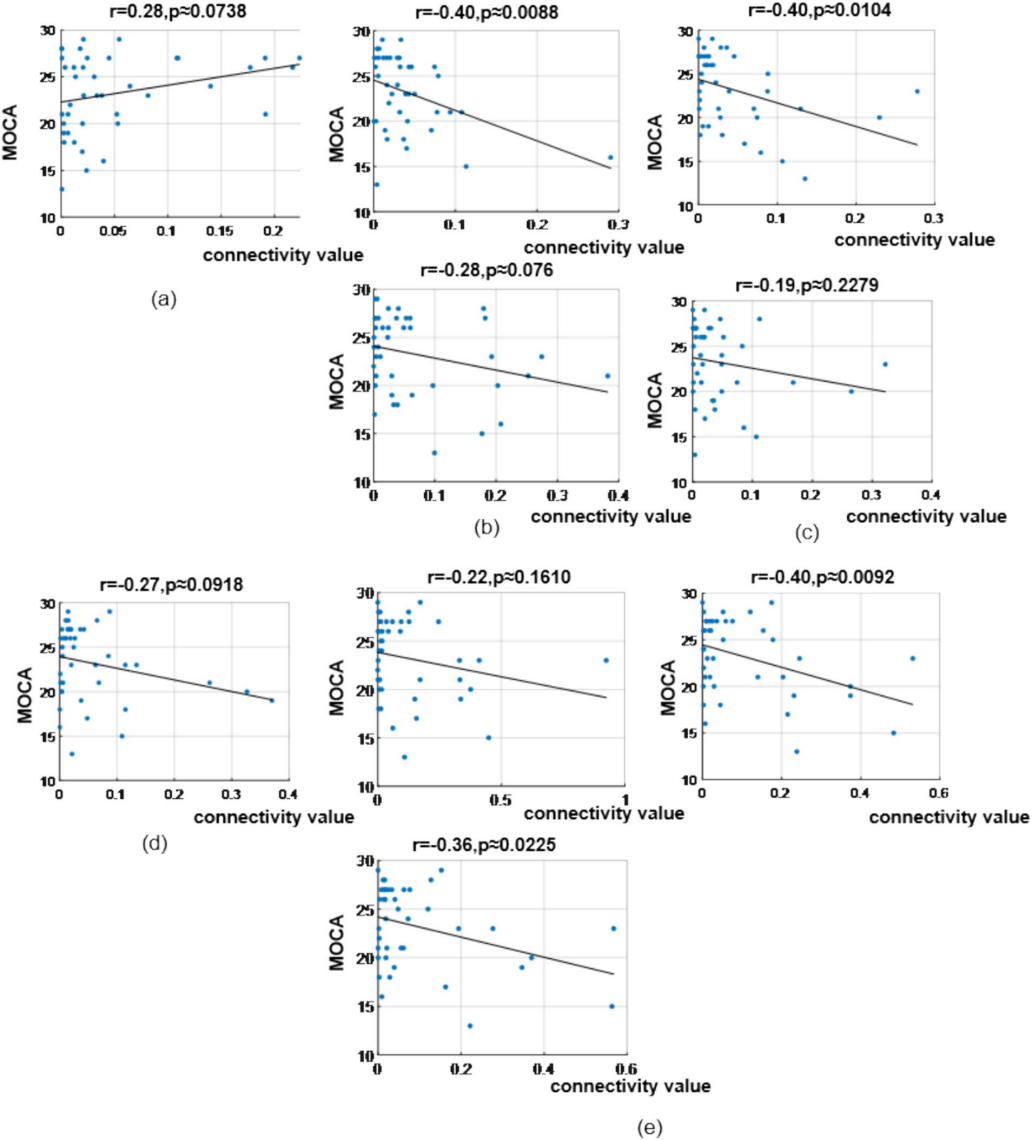
Correlation analysis results between significantly difference brain functional connectivity and MoCA scores in δ-band. **(a)** Correlation between MoCA scores and the prefrontal → prefrontal functional connectivity (AF3> > AF4). **(b)** Correlations between MoCA scores and two prefrontal → parietal functional connectivity (top: Fz> > P3; bottom: F8> > P3). **(c)** Correlations between MoCA scores and two parietal → prefrontal functional connectivity (top: CP5 >> FP2; bottom: CP5 >> F3). **(d)** Correlation between MoCA scores and the parietal → central functional connectivity (P3 >> C3). **(e)** Correlations between MoCA scores and occipital → prefrontal/central/parietal functional connectivity (top-left: PO3 >> F7; bottom-left: PO3 >> C4; top-right: PO3 >> CP5).

To explore the heterogeneity sources of these abnormal connectivity patterns and their associations with behavioral performance, we further analyzed the correlations between significantly different brain functional connectivity and MoCA scores. In the *δ* band, correlation analysis revealed MoCA scores showed a correlation coefficient of (*r* = 0.28, *p* = 0.0738) with the prefrontal-to-prefrontal functional connectivity (AF3 >> AF4); correlation coefficients of (*r* = −0.40, *p* = 0.0088) and (*r* = −0.28, *p* = 0.0760) with the two top-down prefrontal-to-parietal functional connectivity (Fz >> P3, F8 >> P3); correlation coefficients of (*r* = −0.40, *p* = 0.0104) and (*r* = −0.19, *p* = 0.2279) with the two bottom-up parietal-to-prefrontal functional connectivity (CP5 >> FP2, CP5 >> F3); a correlation coefficient of (*r* = −0.27, *p* = 0.0918) with the bottom-up parietal-to-central functional connectivity (P3 >> C3); and correlation coefficients of (*r* = −0.22, *p* = 0.1610), (*r* = −0.36, *p* = 0.0225), and (*r* = −0.40, *p* = 0.0092) with the three bottom-up occipital-to-prefrontal/central/parietal functional connectivity (PO3 >> F7, PO3 >> C4, PO3 >> CP5), as shown in Figure 12.

**Figure 13:**
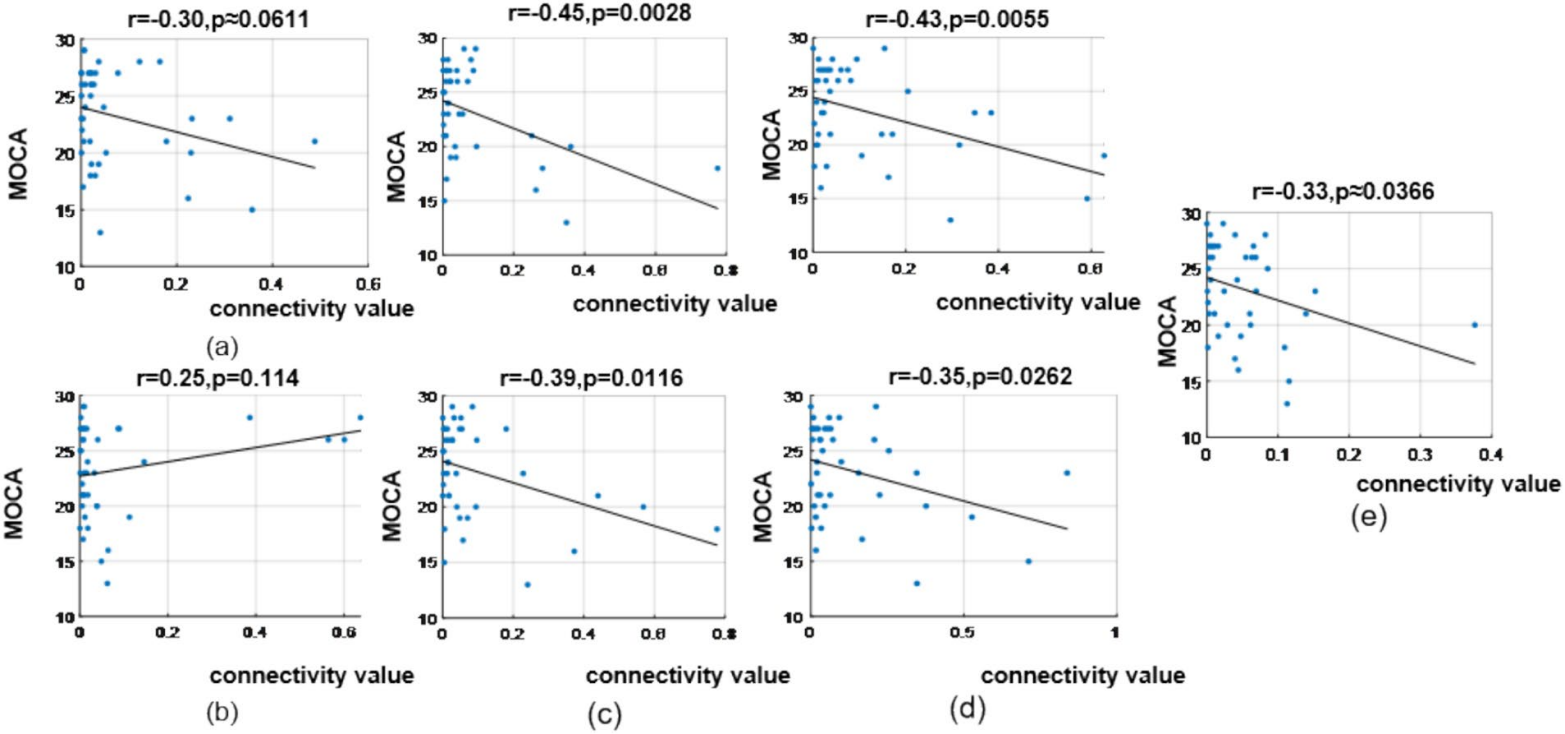
Correlation analysis results between significantly difference brain functional connectivity and MoCA scores in θ-band. **(a)** Correlation between MoCA scores and the prefrontal → prefrontal functional connectivity (F8 >> F7). **(b)** Correlation between MoCA scores and the prefrontal → parietal functional connectivity (FC6 >> P8). **(c)** Correlations between MoCA scores and two temporal → prefrontal functional connectivity (top: T8 >> AF4; bottom: T8 >> F4). **(d)** Correlations between MoCA scores and two occipital → parietal functional connectivity (top: PO3 >> CP5; bottom: PO3 >> P3). **(e)** Correlation between MoCA scores and the parietal → prefrontal functional connectivity (CP5 >> FP2).

In the *θ* band, correlation analysis revealed MoCA scores showed a correlation coefficient of (*r* = −0.30, *p* = 0.0611) with the prefrontal-to-prefrontal functional connectivity (F8 >> F7); a correlation coefficient of (*r* = 0.25, *p* = 0.114) with the top-down prefrontal-to-parietal functional connectivity (FC6 >> P8); correlation coefficient of (*r* = −0.45, *p* = 0.0028) and (*r* = −0.39, *p* = 0.0116) with the two bottom-up temporal-to-prefrontal functional connectivity (T8 >> AF4, T8 >> F4); correlation coefficient of (*r* = −0.43, *p* = 0.0055) and (*r* = −0.35, *p* = 0.0262) with the two bottom-up occipital-to-parietal functional connectivity (PO3 >> CP5, PO3 >> P3); and a correlation coefficient of (*r* = −0.33, *p* = 0.0366) with the bottom-up parietal-to-prefrontal functional connectivity (CP5 >> FP2), as shown in Figure 13.

**Figure 14:**
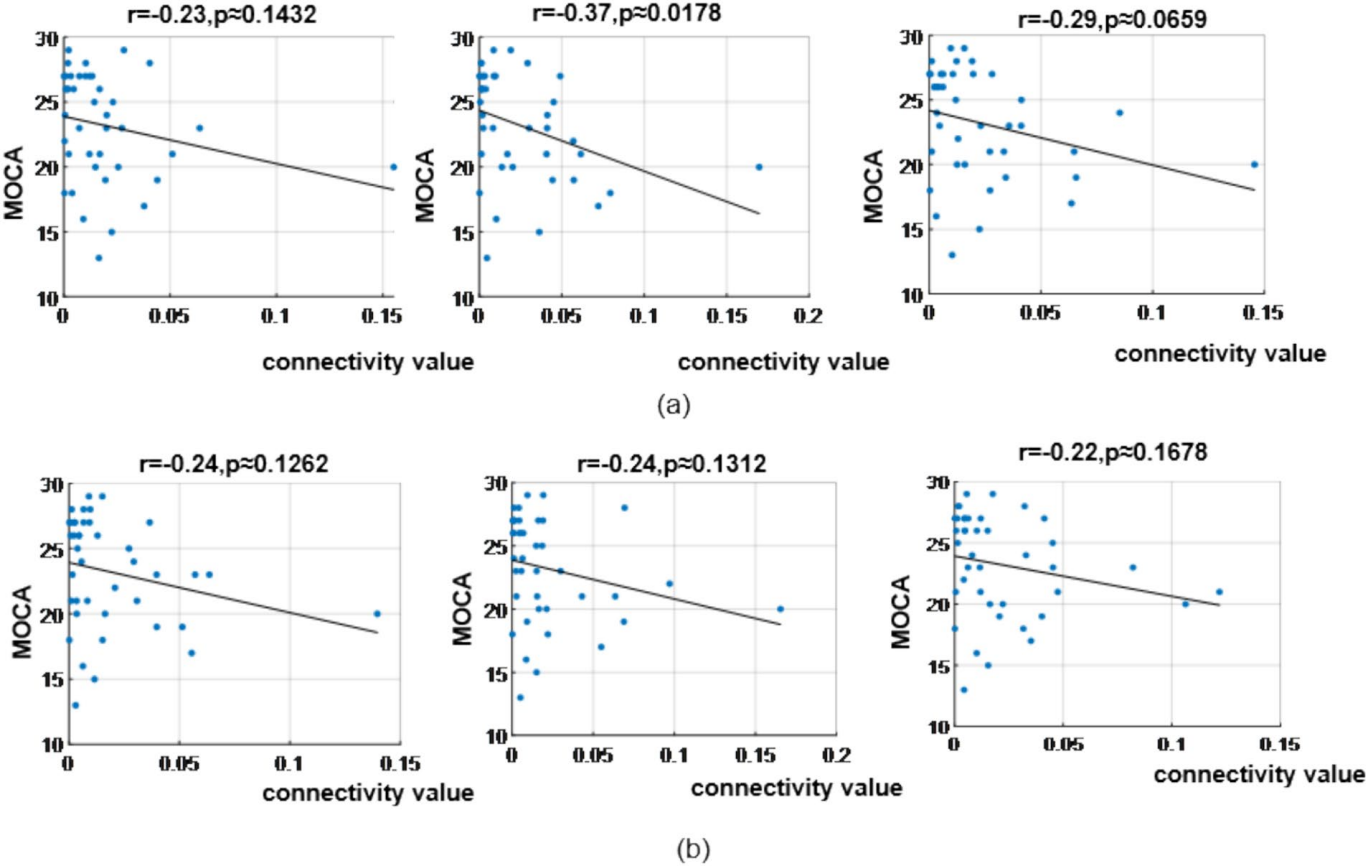
Correlation analysis results between significantly difference brain functional connectivity and MoCA scores in α-band. **(a)** Correlations between MoCA scores and three occipital → prefrontal functional connectivity (Oz >> FP2, Oz >> F8, Oz >> FC6). **(b)** Correlations between MoCA scores and three occipital → parietal functional connectivity (Oz >> CP5, Oz >> CP2, Oz >> P7).

In the *α* band, correlation analysis revealed MoCA scores showed correlation coefficient of (*r* = −0.23, *p* = 0.1432), (*r* = −0.37, *p* = 0.0178) and (*r* = −0.29, *p* = 0.0659) with the three bottom-up occipital-to-prefrontal functional connectivity (Oz >> FP2, Oz >> F8, Oz >> FC6); and correlation coefficient of (*r* = −0.24, *p* = 0.1262), (*r* = −0.24, *p* = 0.1312) and (*r* = −0.22, *p* = 0.1678) with the three bottom-up occipital-to-parietal functional connectivity (Oz >> CP5, Oz >> CP2, Oz >> P7), as shown in Figure 14.

## Discussion

To our knowledge, this was the first study integrating neural oscillations and dynamic functional brain network findings across three primary frequency bands (*δ*, *θ*, α) using PSD and causal dynamic network analysis methods based on resting-state EEG data from MCI and NC. The study revealed abnormal electrophysiological characteristics in the resting-state EEG of MCI patients, uncovering the electrophysiological mechanisms underlying cognitive decline in MCI. Firstly, we discovered that the neural oscillation abnormalities and brain network connectivity abnormalities in the brain regions of MCI were distributed in different frequency bands. Second, our results revealed localized abnormalities in neural oscillations within specific brain regions, coupled with frequency-dependent alterations in dynamic functional connectivity in MCI patients.

Our study demonstrated that MCI exhibits significant differences in *δ*, θ, and α band distributions across brain regions, primarily characterized by widespread δ oscillation enhancement and α oscillation reduction, while θ band differences were observed in fewer electrodes.

### The resting state δ oscillations

δ oscillations during resting state are typically associated with deep rest and sleep states, reflecting a “shutdown” state of the brain. During wakefulness, the presence of *δ* oscillations may be related to attentional lapses or cognitive decline. In our study, a notable increase in delta oscillations among individuals with MCI was primarily observed in the prefrontal and parietal regions (mid-forebrain areas), while such an increase was absent in posterior regions like the occipital lobe, which is consistent with previous research (Babiloni et al., 2013b; Jelic et al., 2000). Interestingly, however, there was a significant decrease in delta oscillations at two electrodes (PO3, PO4) located in the occipital lobe. The prefrontal cortex and parietal cortex (particularly the posterior parietal cortex) play crucial roles in cognitive functions such as working memory, attention, and spatial processing. Increased δ waves in frontal and parietal regions may result from abnormal local neuronal synchronization, potentially caused by amyloid deposition or neurofibrillary tangles (hallmark pathologies of AD) that disrupt excitatory/inhibitory balance. For MCI patients, it’s possible that these changes are linked to early neurodegenerative processes and subsequently result in functional connectivity abnormalities in these important prefrontal–parietal regions. The significant attenuation of δ oscillations in the occipital lobe (across fewer electrodes) in MCI presents an intriguing contrast to the anterior enhancement. Current limited research indicates that patients with AD exhibit enhanced δ oscillations in the occipital lobe during the advanced stages of the disease, which runs counter to our findings (Babiloni et al., 2013a). The occipital lobe, primarily responsible for visual information processing including primary visual cortex (V1) and higher visual areas (Murray et al., 2016). The attenuation of δ oscillations in the occipital lobe may be associated with attentional distraction or cognitive decline, as evidenced by early impairments in visual-related cognitive tasks (such as visual memory) in MCI (Jiang et al., 2024). Furthermore, this could be attributed to the fact that during the MCI stage, while large-scale neuronal death may not have occurred yet, a decline in synaptic plasticity may lead to the suppression of low-frequency activity. Nevertheless, our results should still be interpreted with caution, as the significant attenuation of delta oscillations is currently observed only at a limited number of electrodes.

### The resting state α oscillations

The α oscillations are considered the dominant resting rhythm in awake adults, associated with intelligence, cognition, and memory (Klimesch, 1999). Our study found widespread *α* reduction in MCI patients’ resting-state EEG, particularly in frontal regions, aligning with some previous research (Chae et al., 2020). However, recent studies linking resting-state EEG α rhythms with CSF tau biomarkers reported significantly decreased α source activity in parietal, temporal, and occipital regions in AD-MCI compared to healthy controls and non-AD MCI groups, showing strong negative correlations between CSF phosphorylated tau/total tau levels and posterior α source activity, with weaker correlations to amyloid-β42 levels (Seidu et al., 2024). The discrepancy with our findings of globally reduced α oscillations (predominantly prefrontal) may stem from methodological differences: we collected eyes-open resting-state EEG data, whereas those studies used eyes-closed paradigms. α oscillations are predominantly linked to general attentional processes. The distinct topographical patterns, characterized by a reduction in prefrontal α activity during the eyes-open resting state and a decline in posterior α activity during the eyes-closed resting state, may serve as indicators of state-dependent pathological features in individuals with MCI. Specifically, in the eyes-open condition, the prefrontal cortex employs an *α*-mediated inhibitory mechanism to actively filter out irrelevant visual stimuli (Jensen et al., 2014). In our study, the notable reduction in prefrontal α activity potentially indicates a marked deterioration in the capacity of MCI patients to inhibit distracting information. It is hypothesized that this heightened vulnerability to interference plays a role in the documented decline in cognitive control and memory deficits observed among elderly individuals who do not have cognitive impairment (Voytek and Knight, 2010). Nevertheless, another plausible interpretation is that this reduction in prefrontal *α* during the eyes-open state might be an outward sign of overactive compensatory processes occurring within the prefrontal cortex. For example, in a resting-state magnetic resonance imaging study that compared the default mode network (DMN) activity between patients with amnestic MCI (aMCI) and healthy elderly individuals, it was found that compared with healthy elderly people, patients with aMCI exhibited increased activity in the left prefrontal cortex, the inferior parietal lobule, and the middle temporal gyrus (Qi et al., 2010). This enhanced activity may reflect a compensatory mechanism in the prefrontal cortex aimed at making up for the functional impairment of the DMN. Since *α* waves are typically associated with DMN activity during the resting state, and during task-related states, the prefrontal cortex needs to suppress α waves to activate the working memory network.

### The resting state θ oscillations

Resting-state θ oscillations reflect the neural activity characteristics of the brain in a task-free state and are closely associated with memory encoding, attention regulation, emotional processing, and neural network integration. In our study, both groups showed significant θ oscillation abnormalities in the prefrontal, parietal, and occipital lobes, though the extent was less widespread compared to *δ* and α oscillations. This may be because θ oscillations are more strongly linked to task-related states, where their abnormalities are typically more pronounced—such as in task-related cognitive functions like working memory and attention (Ryan et al., 2021). Additionally, θ oscillation abnormalities are often concentrated in memory- and attention-related brain regions (e.g., prefrontal cortex and hippocampus) (Hasselmo and Stern, 2014), whereas *δ* and α oscillation abnormalities may involve broader areas (e.g., default mode network-related regions) (Sadaghiani et al., 2010; Steriade et al., 1993). The widespread enhancement of δ oscillations in the prefrontal and parietal lobes, coupled with the synchronous reduction of α oscillations in the prefrontal lobe, may reflect both pathological compensation and dysregulation of neuronal activity related to neurodegeneration.

### The δ band functional connectivity

The next issue pertains to differences in network connectivity across frequency bands. In our study, under resting-state conditions, the neural oscillation differences in MCI were distinct from the network connectivity differences. While neural oscillation abnormalities primarily appeared in the δ and α bands, network connectivity differences were most prominent in the δ and θ bands. In the δ band, MCI exhibited significantly enhanced reciprocal functional connectivity between the prefrontal and parietal lobes, as well as strengthened bottom-up functional connectivity from the occipital lobe to the central and parietal regions. Existing studies have yielded heterogeneous results: two studies reported significantly reduced frontal–parietal δ-band functional connectivity in MCI (López et al., 2014; Moretti et al., 2008), one found weakened prefrontal-temporal δ-band functional connectivity (Tóth et al., 2014), while another observed enhanced prefrontal-temporal δ-band functional connectivity (Handayani et al., 2018). The frontoparietal network (FPN) is critical for endogenous attention regulation, cognitive resource allocation, and dynamic network switching (Spreng et al., 2010; Cole et al., 2013). The observed enhancement of δ-band functional connectivity within the frontoparietal network in patients with MCI may suggest functional compensation—an adaptive adjustment to meet cognitive resource demands (Babiloni et al., 2016). However, in the absence of direct behavioral associations (such as task performance indicators) or longitudinal data tracking connectivity over time, this explanation remains speculative. Alternatively, it may stem from neurotransmitter alterations (e.g., reduced acetylcholine leading to decreased cortical excitability and reliance on slower-wave synchronization) (Johansson et al., 2013). Future research needs to incorporate behavioral assessments and longitudinal designs to clarify these mechanisms and identify the adaptive or non-adaptive nature of such connectivity changes. The occipital lobe is central to visual processing, the central region to sensorimotor integration, and the parietal lobe to spatial attention, working memory, and sensory integration. The significantly enhanced bottom-up occipital-to-central/parietal δ-band functional connectivity in MCI. It may reflect abnormal allocation of spatial or attentional resources during rest. This bottom-up pattern suggests that MCI patients excessively rely on low-frequency oscillations for visual/sensory processing during rest (Wang et al., 2007), and this reliance is potentially linked to attention deficits, memory decline, and impaired executive function (Spironelli and Angrilli, 2009), making it a possible marker of early cognitive deterioration.

### The θ band functional connectivity

Dynamic brain network analysis in θ-band revealed significantly enhanced bottom-up functional connectivity including temporal-to-prefrontal, occipital-to-parietal, and parietal-to-prefrontal pathways in MCI. Among prior θ-band resting-state EEG studies, one aligned with our findings (López et al., 2014), reporting enhanced prefrontal-occipital θ-band functional connectivity. While others showed weakened frontal-occipital (Akrofi et al., 2009), weakened frontoparietal (Tóth et al., 2014), or widespread reductions in frontal-occipital, frontal-temporal, frontoparietal, and temporoparietal functional connectivity (Youssef et al., 2021). Previous research findings have shown contradictory results. Moreover, most of the aforementioned studies utilized data obtained during eyes-closed resting state. And none of them employed causal inference methods, thus lacking information on the dynamic flow between brain regions. Consequently, it is difficult to make a relatively objective comparison. θ-band temporal-prefrontal functional connectivity supports memory retrieval and episodic memory integration. Its enhancement in MCI may reflect compensatory efforts to maintain cognitive function via strengthened information transfer, possibly due to hippocampal atrophy or synaptic plasticity decline necessitating stronger oscillatory synchronization (Shankar et al., 2008). Occipital-parietal θ-band functional connectivity typically coordinates visuospatial tasks. Its increase in MCI may reflect patients’ over-reliance on visual information processing, or the parietal lobe’s attempt to compensate for insufficient cognitive resources by increasing synchrony with the sensory cortex. This may be potentially related to dysfunction of the posterior DMN, which leads to a shift of attention from internally oriented states to external stimuli (Greicius et al., 2004; Minoshima et al., 1997). Parietal-prefrontal θ-band functional connectivity underlies goal-directed behavior and cognitive flexibility. Its enhancement in MCI may reflect prefrontal over-regulation of sensory input to offset declining processing speed, possibly due to reduced prefrontal metabolism or dopamine imbalance (Bäckman et al., 2006). θ rhythms depend on precise cholinergic and GABAergic modulation (Jiménez-Balado and Eich, 2021), and degeneration in these systems in MCI may disrupt inter-regional functional connectivity. These widely enhanced bottom-up functional connectivity in MCI suggests MCI may rely more on primary sensory (e.g., occipital) input during rest, with weakened top-down control from higher-order cognitive regions (e.g., prefrontal).

### The α band functional connectivity

Compared to the *δ* and θ frequency bands, the two groups showed smaller yet notable differences in the α frequency band. Specifically, the MCI group exhibited significantly enhanced bottom-up functional connectivity from the occipital lobe to the prefrontal lobe. This contrasts with two prior resting-state studies reporting weakened occipital-prefrontal α connectivity in MCI (López et al., 2014; Handayani et al., 2018), likely due to their use of eyes-closed paradigms and differing network methodologies. As α oscillations are attention-related, the bottom-up occipital-prefrontal flow in MCI may indicate greater dependence on visual input to sustain cognition during rest, reflecting aberrant oscillatory patterns contributing to cognitive decline.

### The limitations

Our study aimed to identify abnormal rhythms and network features characterizing MCI via resting-state EEG analysis, offering insights for future research. However, limitations exist: (1) MCI progression is fluctuating—longitudinal analyses would better capture EEG dynamics; (2) the sample size of this study is relatively small. Although this sample size is sufficient to identify EEG abnormalities with significant effects, it may limit the statistical power for detecting subtle EEG changes and EEG differences. Therefore, the sample size should be expanded in future research to ensure the stability and reproducibility of the results. (3) Although our study identified widespread EEG abnormalities in MCI patients, their clinical heterogeneity warrants emphasis: current diagnostic criteria classify MCI into four core subtypes (single-domain amnestic, multi-domain amnestic, single-domain non-amnestic, and multi-domain non-amnestic) that differ significantly in neuropathological mechanisms, cognitive impairment patterns, and disease progression risks. Notably, this study’s lack of subtype stratification may have obscured critical differences in EEG findings—for instance, reduced prefrontal alpha power could reflect compensatory neural activation secondary to hippocampal dysfunction in MCI, whereas in non-aMCI, this pattern might indicate direct impairment of executive control networks. Therefore, future research should prioritize larger sample sizes with rigorous MCI subtype classification to clarify subtype-specific EEG signatures and their associations with distinct cognitive deficits. (4) Different pre-processing procedures (such as artifact removal and filter settings), as well as network analysis methods (such as insufficient interpretability and lack of standardization), can all lead to variations in results. Although we employed rigorous pre-processing and False Discovery Rate (FDR) correction to minimize bias, residual confounding effects are still difficult to completely eliminate due to the limitation of the small sample size. Moreover, as mentioned above, volume conduction and DTF limitations may introduce spurious connectivity estimates, highlighting the need for more advanced analytical techniques in future research. (5) While cognitive screening tools are valuable for initial assessment and have been widely used in clinical and research settings, relying solely on these methods may limit the diagnostic specificity for MCI. Neuroimaging and biomarker assessments (e.g., Tau protein and Amyloid deposition) offer more objective and sensitive measures that can enhance diagnostic accuracy by providing insights into underlying neuropathological changes associated with MCI and its progression to dementia. The absence of these confirmatory tests in our study increases the potential risk of misclassification. We recognize this limitation and suggest that future studies should consider integrating neuroimaging and biomarker data to improve the reliability and validity of MCI diagnoses. (6) Despite the use of reasonable statistical methods, the small sample size inherently restricts the generalizability of the results and increases the risk of Type I/Type II errors. Future research should prioritize the use of larger datasets and incorporate machine learning/deep learning techniques to construct more robust and reproducible network analysis procedures.

## Conclusion

In the resting state with open eyes, the difference in neural oscillations between MCI and NC mainly occurred in the *δ* and *α* frequency bands, manifested as a decrease the frontal lobe of α oscillations and an increase the frontal lobe and parietal lobe of δ oscillations. However, the main differences in dynamic functional connectivity were in the δ and θ frequency bands. MCI exhibits significantly enhanced functional connectivity between the frontal and parietal lobes, as well as brain functional connectivity between the central and parietal lobes of the occipital lobe from bottom to top in the δ frequency bands. For the θ frequency band, MCI significantly enhances network connectivity between the temporal lobe and frontal lobe from bottom to top, occipital lobe and parietal lobe from top to bottom. The abnormal neural oscillations and integration between brain regions in MCI, as well as the inconsistency in frequency bands and brain regions, further indicate that the neural oscillations and functional integration of brain regions are both related and different.

## Data Availability

The datasets used during the current study are available from the corresponding author on reasonable request.
